# Spontaneous hyphaema secondary to bleeding from an iris vascular tuft in a patient with a supratherapeutic International normalised ratio: case report

**DOI:** 10.1186/s12886-015-0050-y

**Published:** 2015-06-14

**Authors:** Kenneth G. J. Ooi, Rohan Gupta, Sarah B. Wang, Samuel Dance, Armand Borovik, Ian C. Francis

**Affiliations:** Department of Ophthalmology, Prince of Wales Hospital, High Street, Randwick, NSW 2031 Australia; University of Sydney, Camperdown, NSW 2006 Australia; University of New South Wales, Kensington, NSW 2052 Australia

**Keywords:** Iris vascular tuft, hyphaema, supratherapeutic INR, anticoagulation

## Abstract

**Background:**

Iris vascular tufts are rare iris stromal vascular hamartomas. Patients with iris vascular tufts generally remain asymptomatic until presenting with a spontaneous hyphaema or with mild intraoperative pupil margin haemorrhage during anterior segment surgery. This is the first reported case of spontaneous hyphaema from iris vascular tuft related to a documented supratherapeutic International Normalised Ratio as a predisposing factor. At 86 years of age, this patient also represents the oldest documented first occurrence of bleeding from an iris vascular tuft.

**Case presentation:**

An 86 year old Caucasian lady presented with sudden and persisting loss of vision in her right eye, ocular pain and vomiting. She had a supratherapeutic International Normalised Ratio of 3.9 related to Warfarin use. Her intraocular pressure in the right eye was raised at 55 mmHg, with a 1.6 mm hyphaema and multiple iris vascular tufts visible around the entire pupil.

**Conclusion:**

The present case highlights the risk of anticoagulation therapy as a predisposing factor for spontaneous hyphaema and adds to the management considerations for this condition. It also demonstrates the need for Ophthalmologists to be aware of iris vascular tufts as a cause for spontaneous hyphaema, independent of age and systemic associations.

## Background

Iris vascular tufts (IVTs) were first reported by Cobb in 1969 and are otherwise known as iris microhaemangiomas [1]. They are rare iris stromal vascular hamartomas located at the pupillary margin. Their histopathology was first described by the late and renowned Sydney Ophthalmic Pathologist, Dr Marijan Filipic, FRCPA [2]. Using electron microscopy, their lining was described as that of typical endothelial cells with no fenestrations, and joined at their apices. Leakage on angiography is thought to be explained by breaks within the endothelial cells [2].

IVTs typically remain asymptomatic until presenting as a spontaneous hyphaema, with blurred vision, and occasionally erythropsia and raised intraocular pressure (IOP). Recurrent episodes of hyphaema are known to occur [2, 3]. They may also be seen as tiny haemorrhages on the pupil margin during otherwise routine anterior segment surgery. If the hyphaema clears within minutes or hours, the transient visual loss may be misdiagnosed. Indeed, disorders such as amaurosis fugax, mild iritis, or Posner-Schlossman syndrome may be considered.

IVTs occur most commonly between the ages of 42 to 80 years, with no racial or gender bias, and can occur bilaterally [4, 5]. Systemic associations are occasionally present, including diabetes mellitus, chronic obstructive pulmonary disease and myotonic dystrophy [6, 7]. An association with idiopathic juxtafoveal retinal telangiectasia has also been reported [8].

This case documents, for the first time, bleeding from IVTs in association with a supratherapeutic International Normalised Ratio (INR). It is possible the patient’s emergent presentation may ultimately have saved her life by preventing systemic haemorrhage. This paper was written in accordance with the Declaration of Helsinki 1995.

## Case presentation

An 86 year old Caucasian lady presented to the Emergency Department (ED) of a tertiary referral teaching hospital with sudden and persisting loss of vision in her right eye over eight hours. The ED team reported that a hyphaema was present with ‘bleeding through the pupil’. The patient described reduced vision after waking that morning, which progressed during the day. She reported severe pain (6/10) and aching in the right eye, which extended ipsilaterally down to her neck and was associated with dry retching every 30 min. She denied any previous similar episodes.

She was on Warfarin for recurrent pulmonary emboli and had recently been prescribed intravenous Ceftriaxone and subsequent oral Cefaclor for cellulitis of her right leg. Her general health had been complicated by a cerebrovascular accident 30 years earlier following a ventriculoperitoneal shunt revision originally performed for pseudotumour cerebri. She had treated hypothyroidism and hypertension.

Her right visual acuity was light perception and left was 6/9. The right pupil response to light was sluggish, but due to her long-standing Parinaud’s syndrome secondary to the stroke, pupillary assessment was challenging. However, there was no evidence of a relative afferent pupil defect, nor indeed of an indirect relative afferent pupil defect. In the right eye there was a 1.6 mm hyphaema. Her IOP was 55 mmHg, with slight corneal microcystic oedema. There was no evidence of pupillary block and her anterior chamber angles were open at grade 2. There was no evidence of primary uveal melanoma. The left eye was normal, except for a small area of temporal foveal atrophy.

B-scan ultrasound was normal with no evidence of vitreous haemorrhage or mass lesions identified. An iris fluorescein angiogram was contemplated and would have supported a more definitive diagnosis of iris microhaemangioma but was deemed clinically unnecessary at the time. A computed tomography angiogram of the brain, conducted in ED to investigate the patient’s symptoms, with a view to excluding internal carotid artery stenosis, was also normal. Full blood count, liver function tests, blood sugar level and HbA1C were normal. Coagulation studies revealed a supratherapeutic INR of 3.9 and electrolyte analysis revealed elevated serum potassium of 5.9 mEq/L, both of which were deemed iatrogenic secondary to her recent oral antibiotic use.

**Fig. 1:**
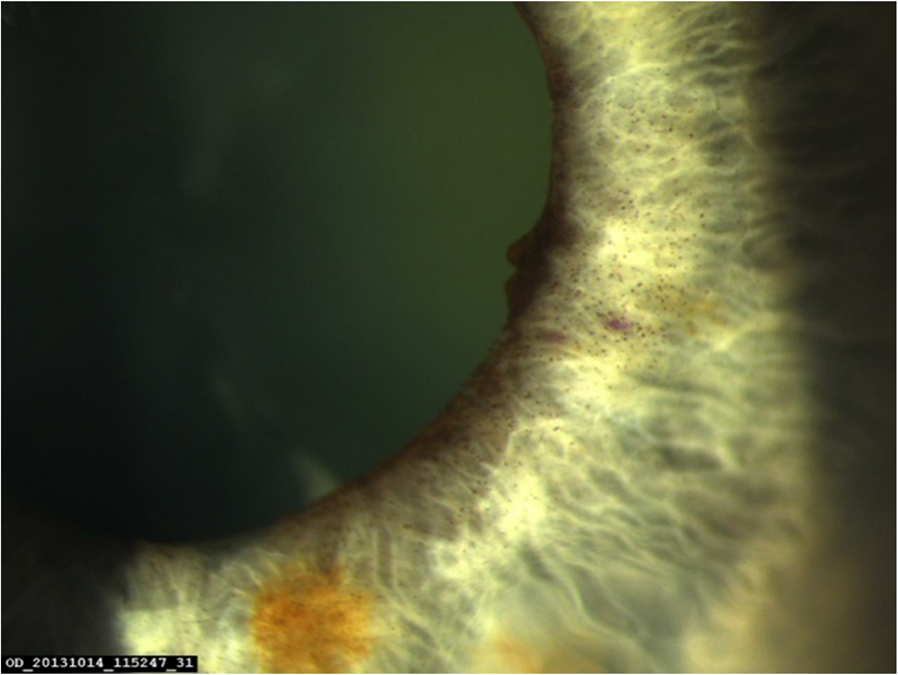
Iris Microhaemangiomas. Iris Microhaemangiomas on right pupil visualised after clot retraction

A 23-gauge needle anterior chamber paracentesis was performed temporally. The intraocular pressure was lowered to 29 mmHg, and statim dilatation was carried out with guttae (G.) Tropicamide 1 %. As the pupillary diameter increased, clot retraction occurred with clearing of the visual axis, immediate improvement in right eye visual acuity to 6/60, and demonstration of the ipsilateral IVTS (Fig. 1). At this point, the IVTS in the left eye also became evident. Right fundus examination demonstrated a normal optic nerve head with a cup:disc ratio of 0.2, and with a small area of central foveal atrophy.

The patient was commenced on hourly G. Prednisolone Acetate 1 %, G. Atropine 1 % twice daily, and a combination of Brinzolamide, Timolol, Latanoprost and Brimonidine. With the advice of the Haematologists, she was administered 1 mg of Vitamin K intravenously in order to normalise her INR. With advice from the Nephrologists, 30 g of Resonium was administered orally to reduce her serum potassium.

Her hyphaema resolved completely, and after 48 h her right visual acuity had improved to 6/12. Subsequent scanning laser polarimetry of the retinal nerve fibre layer and automated achromatic perimetry testing were within normal limits.

Given the absence of any significant trauma and lack of systemic associations, the patient’s spontaneous hyphaema was attributed to her IVTs with supratherapeutic INR as a predisposing factor. Her recent oral cephalosporin usage for lower limb cellulitis was implicated as causal for her high INR. It is known that these antibiotics decrease the absorption and alter the metabolism of vitamin K, thus augmenting the effects of Warfarin. Haematology review deemed her risk of further clotting episodes to be too high to stop warfarinisation. Counselling was given to the patient on the association of an increased, and potentially life-threatening INR, and propensity for further IVT bleeding.

## Conclusion

Most patients with IVTs remain undiagnosed, and of those patients in whom the condition is detected, few receive any treatment. Conservative management of IMHs in the initial instance is more than appropriate, as most patients develop only a single, self-limiting episode of hyphaema. Argon laser photocoagulation of the iris is reserved for cases complicated by recurrent hyphaema. Several treatment sessions may be needed, although generally only one is required. In even more rare cases, iris fluorescein angiography can be useful in locating precisely the true extent of the leaking IVTs [8]. In addition to argon laser photocoagulation, topical and systemic steroids can be used to induce IVT regression or hasten spontaneous shrinkage.

The patient admitted to typically sleeping on her right side, and may have encountered pressure on her eye during sleep. This may have caused her to undergo mild iris trauma with a secondary ‘traumatic’ hyphaema [9], which may have subsequently worsened or re-bled with eye rubbing. Whether spontaneous or secondary to mild trauma, the degree of anticoagulation in this patient was likely to have contributed significantly to the magnitude of the hyphaema, especially given the absence of any systemic associations, the tight control of other risk factors (hypertension) and the fact that there were no previous episodes of hyphaema with a normally well-controlled INR.

In conclusion, this is a novel case of an elderly lady with IVTs and subsequent spontaneous hyphaema which in our opinion was likely related to over-anticoagulation and possibly aggravated by sleeping on the ipsilateral side. The age of our patient at 86 years was also unusual and represents the oldest documented first occurrence of bleeding from an IVT. Her presentation underscores the need for the Ophthalmologist to be aware of IVTs as a cause for spontaneous hyphaema, independent of age and systemic associations thereof. Finally, this case not only adds to the management considerations for this condition, but also demonstrates, as with unexpected bleeding from any site, that the possibility of the patient being over-anticoagulated should always be considered.

## Consent

Written informed consent was obtained from the patient for publication of this Case Report and accompanying images. A copy of the written consent is available for review by the Editor of this journal.

## Abbreviations

IMH: Iris microhaemangioma
INR: International Normalised Ratio
IOP: Intraocular Pressure
ED: Emergency Department
G: Guttae
